# Exploratory Analysis of Biomarkers and Treatment Outcomes from the COLUMBUS Study in BRAF V600E/K–Mutant Advanced or Metastatic Melanoma

**DOI:** 10.1158/1078-0432.CCR-25-3262

**Published:** 2026-01-15

**Authors:** Reinhard Dummer, Shibing Deng, Tao Xie, Nuzhat Pathan, Hedieh Saffari, Caroline Robert, Ana Arance, Jan Willem B. de Groot, Claus Garbe, Helen J. Gogas, Ralf Gutzmer, Ivana Krajsová, Gabriella Liszkay, Carmen Loquai, Mario Mandala, Dirk Schadendorf, Naoya Yamazaki, Paolo A. Ascierto, Craig B. Davis, Khyati Shah, Phineas Hamilton, Alessandra di Pietro, Keith Flaherty

**Affiliations:** 1 University Hospital Zurich, Zurich, Switzerland.; 2 Pfizer, La Jolla, California.; 3 Pfizer, South San Francisco, California.; 4 Gustave Roussy and Paris-Saclay University, Villejuif, France.; 5 Hospital Clinic of Barcelona and IDIBAPS, Barcelona, Spain.; 6 Isala Oncology Center, Zwolle, Netherlands.; 7 University Hospital Tübingen, Tübingen, Germany.; 8 National and Kapodistrian University of Athens, Athens, Greece.; 9 Department of Dermatology, Ruhr University Bochum, Minden, Germany.; 10 General Teaching Hospital in Prague, Prague, Czech Republic.; 11 National Institute of Oncology, Budapest, Hungary.; 12 Department of Dermatology, Fachklinik Hornheide, Muenster, Germany.; 13 University of Perugia, Perugia, Italy.; 14 University Hospital Essen, West German Cancer Center and German Cancer Consortium, Partner Site Essen, Essen, Germany.; 15 National Center for Tumor Diseases (NCT) West, Campus Essen, and Research Alliance Ruhr, Research Center One Health, University Duisburg-Essen, Essen, Germany.; 16 National Cancer Center Hospital, Tokyo, Japan.; 17 Melanoma Unit, Cancer Immunotherapy and Innovative Therapies, Istituto Nazionale Tumori IRCCS Fondazione Pascale, Naples, Italy.; 18 Pfizer, Milan, Italy.; 19 Massachusetts General Hospital Cancer Center, Boston, Massachusetts.

## Abstract

**Purpose:**

Treatment with encorafenib ± binimetinib is associated with improved survival versus vemurafenib in patients with BRAF V600E/K–mutant advanced melanoma. We retrospectively analyzed genomic and transcriptomic data from the phase III COLUMBUS trial to identify molecular correlates of benefit with encorafenib ± binimetinib.

**Experimental Design:**

In COLUMBUS, patients with BRAF V600E/K–mutant locally advanced, unresectable, or metastatic melanoma (*n* = 921) were randomized to receive encorafenib plus binimetinib, encorafenib, or vemurafenib. We used whole-exome sequencing (*n* = 666), whole-transcriptome sequencing (RNA sequencing; *n* = 514), and assessment of circulating tumor DNA (ctDNA) at baseline (*n* = 336) and on treatment (cycle 2 day 1, *n* = 184) to evaluate biomarker associations with progression-free and overall survival.

**Results:**

Survival benefits with encorafenib plus binimetinib versus vemurafenib were greatest in patients with higher tumor mutational burden (TMB) and those with evidence of tumor immune infiltration (i.e., higher cytolytic score, PD-L1 expression, or IFNγ gene signature scores). Clustering of gene expression profiles identified three tumor subgroups, including an “immune” subgroup associated with improved survival. Detection of *BRAF* V600 alterations in baseline ctDNA was associated with shorter survival; clearance of *BRAF* V600 alterations at cycle 2 day 1 was associated with improved survival across arms.

**Conclusions:**

The greatest benefits of encorafenib plus binimetinib were observed in patients with evidence of high TMB and/or tumor-immune infiltration, suggesting potential immune contributions to efficacy, which were not observed with vemurafenib. *BRAF* V600 detectability in ctDNA seems to have utility as a marker of prognosis and response in this population.

**Translational Relevance**

The phase III COLUMBUS trial previously demonstrated that encorafenib plus binimetinib and encorafenib monotherapy improved progression-free survival (PFS) and overall survival (OS) versus vemurafenib in patients with BRAF V600E/K–mutant locally advanced, unresectable, or metastatic melanoma. Patients were either treatment-naïve or had progressed after first-line immunotherapy and had not received prior BRAF or MEK inhibitors. The combination of encorafenib plus binimetinib provided the best clinical outcome for this patient population. In this study, we found that the greatest survival benefits with encorafenib plus binimetinib were seen in patients with evidence of preexisting immune cell infiltration and higher tumor mutational burden. Associations between detectability of *BRAF* alterations in circulating tumor DNA (ctDNA) at baseline and during treatment and PFS and OS suggest ctDNA as a potential marker of prognosis and benefit in BRAF V600E/K–mutant advanced melanoma.

## Introduction

*BRAF* V600 mutations occur in approximately 50% of patients with cutaneous melanoma (1–3). COLUMBUS (NCT01909453) was a randomized, two-part, multicenter, open-label, phase III study that assessed the safety and efficacy of the BRAF inhibitor encorafenib plus the MEK inhibitor binimetinib versus monotherapy with the BRAF inhibitors vemurafenib or encorafenib in patients with BRAF V600E/K–mutant locally advanced, unresectable, or metastatic melanoma (4). COLUMBUS led to the approval of the combination of encorafenib plus binimetinib in this population based on improved progression-free [PFS; 14.9 vs. 7.3 months; hazard ratio (HR), 0.51] and overall survival (OS; 33.6 vs. 16.9 months; HR, 0.67) versus vemurafenib (2, 4–8).

Despite the demonstrated benefits of encorafenib plus binimetinib, primary and acquired resistance mechanisms remain significant clinical challenges (9). Since the approval based on COLUMBUS, resistance mechanisms and validated novel biomarkers for treatment optimization have been elucidated. Demonstrated mechanisms of resistance include alternate activation of the MAPK pathway through activating mutations in *NRAS*, *KRAS*, or *MAP2K1* and activating mutations in the PI3K pathway involving *AKT*, *PTEN*, and *MTOR* (10). Beyond genetic alterations, single-cell analysis has revealed that drug-tolerant transcriptional states can arise in response to treatment (11), whereas specific cancer cell phenotypic or tumor microenvironment (TME) features may also confer primary resistance (12). Examples include *MITF*, which exhibits dual resistance functions through *MITF*-high/*AXL*-low versus *MITF*-low/*AXL*-high phenotypic switching (13), and *POSTN*, which fosters resistance by promoting emergence of tumor cell–protective macrophages that directly shield melanoma cells from treatment-induced cell death (12). Importantly, signatures of immune infiltration have been associated with increased benefit from BRAF plus MEK inhibition, with treatment promoting enhanced CD8^+^ T-cell infiltration and improved antigen presentation (14). Circulating tumor DNA (ctDNA) has also emerged as a biomarker for monitoring treatment response, detecting resistance an average of 3.6 months earlier than conventional imaging (15). Although current clinical practice guidelines acknowledge biomarker importance, comprehensive biomarker evaluation beyond *BRAF* mutation testing has not yet been incorporated into standard practice recommendations (8).

In this study, we evaluate whether the addition of binimetinib to encorafenib led to increased benefit in specific biomarker molecularly defined subgroups and whether encorafenib alone confers increased benefit versus vemurafenib in specific subgroups. This leveraged analysis of long-term 7-year PFS and OS, along with comprehensive genomic and transcriptomic profiling of patients treated in COLUMBUS, to better understand the correlates of benefit with BRAF inhibition ± MEK inhibition in BRAF V600E–mutant melanoma. Analyses of whole-exome sequencing (WES) and whole-transcriptome sequencing [RNA sequencing (RNA-seq)] were performed, as was ctDNA profiling of somatic mutations at baseline and on treatment, representing the largest cohort of *BRAF*-mutant melanoma samples comprehensively characterized to date.

## Materials and Methods

### COLUMBUS study

COLUMBUS (NCT01909453) is a randomized, two-part, multicenter, open-label, phase III study (4, 5, 16–18). The COLUMBUS study design, primary analysis, and 5- and 7-year updates have been published previously (4, 5, 17, 18). Briefly, eligible patients were treatment-naïve or had progressed after first-line immunotherapy, had an Eastern Cooperative Oncology Group performance status of 0 or 1, and had not had prior therapy with a BRAF inhibitor and/or MEK inhibitor. In COLUMBUS part 1, 577 patients were randomized 1:1:1 to receive encorafenib 450 mg once daily plus binimetinib 45 mg twice daily, encorafenib 300-mg once-daily monotherapy, or vemurafenib 960-mg twice-daily monotherapy. In COLUMBUS part 2, 344 patients were randomized 3:1 to receive encorafenib 300 mg once daily plus binimetinib 45 mg twice daily or encorafenib 300 mg once-daily monotherapy (Supplementary Fig. S1).

This exploratory biomarker analysis was performed in enrolled patients with available samples for WES, RNA-seq, and/or ctDNA analyses. The study protocol was approved by independent ethics committees or site institutional review boards. The conduct of the study conformed to Good Clinical Practice guidelines and the ethical requirements outlined in the Declaration of Helsinki. Written informed consent was obtained from all patients before screening.

### Endpoints

This exploratory biomarker analysis evaluated genomic and transcriptomic correlates of the patients’ clinical outcomes. PFS was assessed by blinded independent central review and was defined as the time from the date of randomization to the date of the first documented disease progression or death due to any cause, whichever occurred first. OS was defined as the time from the date of randomization to the date of death due to any cause. PFS and OS data from the 7-year analysis (data cutoff: January 13, 2023) were analyzed by treatment groups and the presence of specific genetic alterations or transcriptomic features at baseline.

Data from COLUMBUS parts 1 and 2 were pooled for the encorafenib plus binimetinib arm (part 1, encorafenib 450 mg once daily; part 2, encorafenib 300 mg once daily) and for encorafenib monotherapy (300 mg once daily in both parts) to increase the power for biomarker analysis.

### WES, whole-transcriptome sequencing, and analysis

Baseline tumor samples were analyzed using the ACE ImmunoID NeXT WES/RNA-seq assays (Personalis Inc.). WES and RNA-seq of formalin-fixed, paraffin-embedded (FFPE) blocks or slide samples were performed by Personalis using the ImmunoID NeXT platform and sequenced on the Illumina NovaSeq Sequencing system. DNA and RNA were dual-isolated from FFPE blocks or slides using the AllPrep DNA/RNA FFPE Kit (Qiagen). WES capture was performed using SureSelect Clinical Research Exome v.2 (Agilent Technologies) according to the manufacturer’s recommendations. Additional supplementation with Personalis proprietary target probes was performed to enhance coverage in difficult-to-sequence regions within sets of biomedically and medically relevant genes. For WES, Personalis proxy normal and custom filters were used to remove germline variants. Matched normal controls were unavailable; hence, variants were filtered for presumptive somatic status based on Personalis’ internal control pipeline. Somatic mutations with variant allele frequency (VAF) above 0.05 were reported. For RNA-seq, Personalis provided gene-level read counts and transcript per million (TPM)-normalized gene expression values. Samples with fewer than 25M mapped reads were excluded from further analyses.

Analyses of gene expression were based on a log_2_ (x + 1) transformation of TPM values. Unsupervised clustering and estimation of tumor cell type compositions using K-means clustering and xCell were performed to identify candidate molecular subtypes and potential cell type contributions to subtypes, respectively (19). Clustering on gene expression and global analyses of gene expression with outcomes were performed after filtering genes to exclude those with a standard deviation in log expression <1 across samples.

### Biomarker assessments

Tumor mutational burden (TMB) [mutations (mut)/Mb] was determined by Personalis’ proprietary bioinformatics pipeline based on WES. The cytolytic score was determined as the average log-transformed *GZMA* and *PRF1* TPM (20). PD-L1 (encoded by *CD274*) expression levels were evaluated as log TPM. The GO-BP IFNγ gene expression signature was used to calculate an IFNγ gene expression score as the mean of log TPM values of signature genes. The PI3K pathway was considered mutated if mutations were detected in *PIK3CA*, *PTEN*, *AKT* (*AKT1*, 2, and 3), or *MTOR genes*. *MITF* and *AXL* gene expression levels were evaluated from log TPM. For patient stratification for TMB, cytolytic score, PD-L1 expression, IFNγ signature, and *ERBB2* expression, biomarker-high subgroups were defined as those with biomarker levels above the median for the population, and biomarker-low subgroups as those with levels at or below the median. *BRAF* mutation status (V600E or V600K) was taken from local test results reported at patient enrollment; in one case in which both *BRAF* V600E and V600K were reported, V600K was considered to be present.

### ctDNA analysis

Plasma samples were collected for ctDNA analysis at screening, cycle 1 day 1 (C1D1, and C2D1, with the C1D1 values taken as baseline. Samples were sequenced using the GuardantOMNI assay (Guardant Health Inc.) to evaluate somatic alterations. Variants were detected and reported based on a threshold of ≥0.01% VAF, as determined by Guardant’s proprietary bioinformatics pipeline. As study patients were known to have *BRAF* V600 alterations, *BRAF* V600 alteration VAF was used as the metric for ctDNA burden in the analysis.

### Statistical analysis

Median durations of follow-up for PFS and OS were estimated by reverse Kaplan–Meier analysis. HR were estimated using Cox proportional hazard regression models and presented along with 95% confidence intervals (CI). Cluster analysis was performed using the K-means clustering method, with the optimal number of k clusters determined by the average silhouette width. Recursive partitioning was conducted based on preselected predictor variables using the *rpart* R package, including only cases with complete data for each variable. Unless otherwise indicated, adjustments correcting for multiple testing were not performed, and all results should thus be interpreted as hypothesis-generating. All analyses were performed using R v4.1.0 or above (RRID: SCR_001905).

## Results

### Study cohort

In the COLUMBUS study, a total of 921 patients with locally advanced unresectable or metastatic BRAF V600E/K–mutant melanoma were randomized. In part 1 of COLUMBUS, 577 patients were randomized to receive encorafenib plus binimetinib (*n* = 192), vemurafenib monotherapy (*n* = 191), or encorafenib monotherapy (*n* = 194). In COLUMBUS part 2, an additional 344 patients were randomized to receive encorafenib plus binimetinib (*n* = 258) or encorafenib monotherapy (*n* = 86; Supplementary Fig. S1). Overall, 668 patients had WES or RNA-seq data (Supplementary Fig. S2); 666 patients had WES, 514 patients had RNA-seq, 336 patients had baseline ctDNA, and 184 patients had on-treatment ctDNA successfully profiled. Baseline characteristics by biomarker status are shown in Table 1 for tumor tissue biomarkers and were similar between the biomarker set and the overall study population.

**Table 1.** Baseline patient characteristics by biomarker status.

Variable	Overall	Biomarker set		*P* value
	(*N* = 921)	No (*n* = 253)	Yes (*n* = 668)	
Age, years	​	​	​	​
Median (range)	56 (47–66)	55 (46–65)	57 (47–66)	0.2
Sex, *n* (%)	​	​	​	​
Female	392 (43)	105 (42)	287 (43)	​
Male	529 (57)	148 (58)	381 (57)	​
Race, *n* (%)	​	​	​	0.022
Missing	1 (0.1)	1 (0.4)	0 (0)	​
Asian	41 (4.5)	6 (2.4)	35 (5.2)	​
Black	1 (0.1)	1 (0.4)	0 (0)	​
White	838 (91)	229 (91)	609 (91)	​
Native American	4 (0.4)	1 (0.4)	3 (0.4)	​
Other	9 (1)	5 (2)	4 (0.6)	​
Unknown	27 (2.9)	10 (4)	17 (2.5)	​
Treatment, *n* (%)	​	​	​	​
Untreated	10 (1.1)	10 (4)	0 (0)	​
Encorafenib (part 1)	192 (21)	72 (28)	120 (18)	​
Encorafenib (part 2)	84 (9.1)	6 (2.4)	78 (12)	​
Encorafenib 300 mg + binimetinib	257 (28)	33 (13)	224 (34)	​
Encorafenib 450 mg + binimetinib	192 (21)	76 (30)	116 (17)	​
Vemurafenib	186 (20)	56 (22)	130 (19)	​
*BRAF* screening, *n* (%)	​	​	​	0.4
Missing	2 (0.2)	1 (0.4)	1 (0.1)	​
V600E	815 (88)	227 (90)	588 (88)	​
V600K	104 (11)	25 (9.9)	79 (12)	​

### TMB and benefit from encorafenib ± binimetinib versus vemurafenib

**Figure 1.**
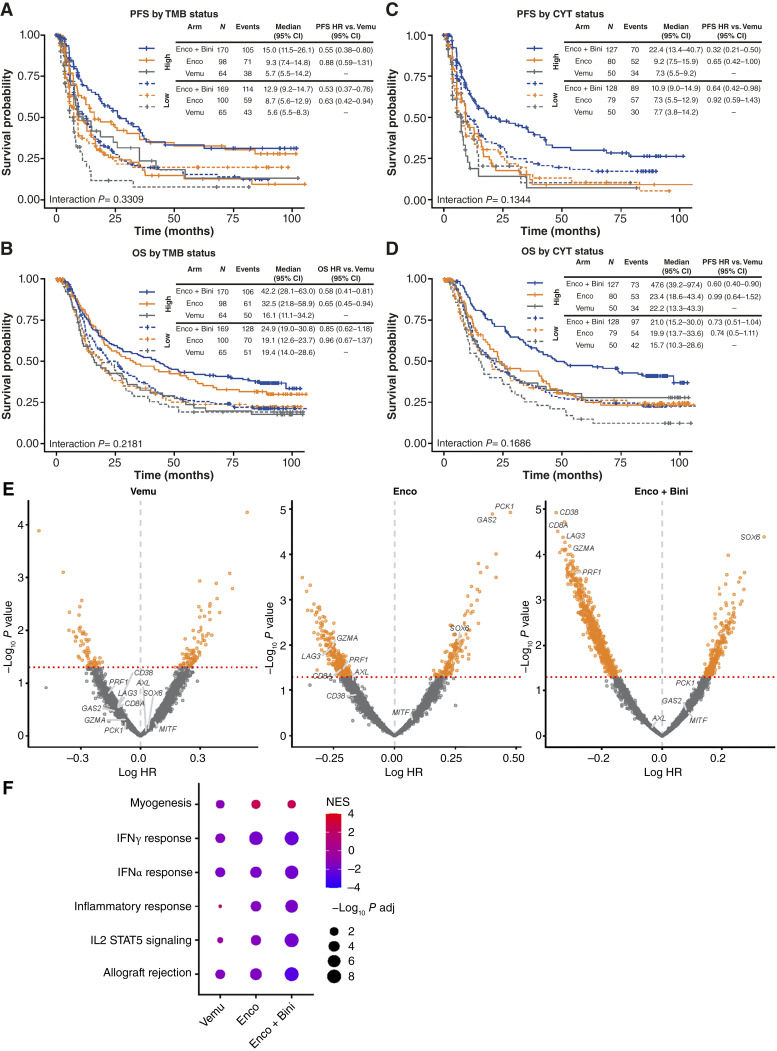
PFS and OS for encorafenib plus binimetinib or encorafenib vs. vemurafenib by (**A** and **B**) TMB and (**C** and **D**) cytolytic score. **E,** Volcano plots of univariable gene expression associations with OS in each arm based on *z*-scored gene expression values. **F,** Gene-set enrichment analysis of gene expression associations against hallmark gene signatures; signatures with greatest average NES across arms shown. Bini, binimetinib; BM, biomarker; CYT, cytotoxic score; Enco, encorafenib; NES, normalized enrichment scores; Vemu, vemurafenib.

The range for TMB was 5.28 to 33.56 mut/Mb. Patients were considered to have low or high baseline TMB based on the median TMB value among patients profiled in the study (8.64 mut/Mb). Encorafenib plus binimetinib resulted in a PFS benefit versus vemurafenib regardless of the TMB group [HR, 0.55 (95% CI, 0.38–0.80) for high TMB and HR, 0.53 (95% CI, 0.37–0.76) for low TMB]; however, patients in the TMB-high group had a longer PFS versus patients in the TMB-low group for both treatment arms [HR, 0.71 (95% CI, 0.55–0.93) with encorafenib plus binimetinib (high vs. low TMB) and HR, 0.74 (95% CI, 0.48–1.15) with vemurafenib (high vs. low TMB)]. In contrast, encorafenib monotherapy provided an improved PFS benefit versus vemurafenib in the TMB-low group [HR, 0.63 (95% CI, 0.43–0.94)] but not in the TMB-high group [HR, 0.88 (95% CI, 0.59–1.31)]. No difference in PFS was observed between patients in the TMB-high and TMB-low groups treated with encorafenib monotherapy [HR, 1.01 (95% CI, 0.71–1.42); Fig. 1A].

Encorafenib plus binimetinib or encorafenib monotherapy resulted in a greater OS benefit versus vemurafenib in the TMB-high group compared with the TMB-low group (Fig. 1B). OS was improved with both encorafenib plus binimetinib [HR, 0.58 (95% CI, 0.41–0.81)] and encorafenib monotherapy [HR, 0.65 (95% CI, 0.45–0.94)] versus vemurafenib in the TMB-high group, with no OS benefits observed versus vemurafenib observed in the TMB-low group.

Additionally, TMB was elevated in patients with *BRAF* V600K (*n* = 79) versus *BRAF* V600E (*n* = 586) mutations [median, 10.60 vs. 8.47 mut/Mb; *P* < 0.001 (*t* test)]; however, adjusting for *BRAF* mutation type in models did not affect the increased OS benefit of encorafenib and encorafenib plus binimetinib versus vemurafenib observed in the TMB-high group (adjusted HR, 0.65 and 0.58, respectively).

### Transcriptional correlates of PFS and OS with encorafenib plus binimetinib or encorafenib monotherapy versus vemurafenib

The range for cytolytic scores was 0.12 to 6.87 log_2_ TPM. Patients were considered to have low or high cytolytic scores based on the median score values. For patients in the high cytolytic score group, encorafenib plus binimetinib resulted in greater PFS (Fig. 1C) and OS (Fig. 1D) benefits versus vemurafenib compared with patients in the low cytolytic score group [PFS HR, 0.32 (95% CI, 0.21–0.50) for high cytolytic score and 0.64 (95% CI, 0.42–0.98) for low cytolytic score; OS HR, 0.60 (95% CI, 0.40–0.90) for high cytolytic score and 0.73 (95% CI, 0.51–1.04) for low cytolytic score]. Lower PFS or OS benefits were observed with encorafenib monotherapy versus vemurafenib [PFS HR, 0.65 (95% CI, 0.42–1); OS HR, 0.99 (95% CI, 0.64–1.52)]. In the low cytolytic score group, although PFS was significantly longer with encorafenib plus binimetinib versus vemurafenib [HR, 0.64 (95% CI, 0.42–0.98)], no PFS benefit was observed with encorafenib monotherapy versus vemurafenib [HR, 0.92 (95% CI, 0.59–1.43)]. Similar trends were observed when grouping patients by PD-L1 (*CD274*) expression as well as the IFNγ gene signature score based on their median values (Supplementary Fig. S3A–S3D), with PFS and OS benefits with encorafenib plus binimetinib and encorafenib monotherapy versus vemurafenib numerically greatest in patients with high PD-L1 (*CD274*) expression or high IFNγ. As expected, these RNA-derived metrics of tumor immune infiltration were highly correlated across patients (*r* > 0.75; Supplementary Fig. S3E).

Taken together, these analyses suggest the greatest treatment benefit from encorafenib plus binimetinib versus vemurafenib for patients with evidence of potential antitumor immune responses that included higher TMB, cytolytic score, PD-L1 expression level, and IFNγ gene expression signature scores (Fig. 1; Supplementary Fig. S3). Although qualitatively similar effects were generally observed for encorafenib monotherapy versus vemurafenib, these effects were generally not as strong and did not always reach statistical significance, suggesting a greater importance of immune contexture to the effects of encorafenib plus binimetinib than encorafenib alone.

**Figure 2.**
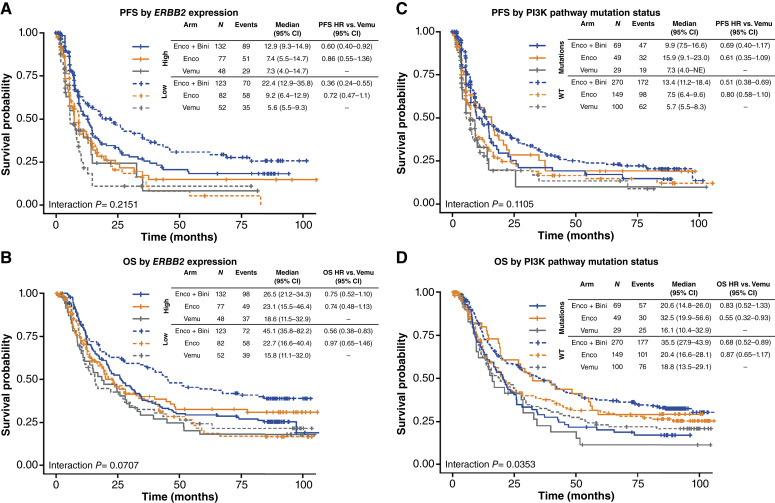
PFS and OS for encorafenib plus binimetinib or encorafenib vs. vemurafenib by (**A** and **B**) *ERBB2* expression level and (**C** and **D**) PI3K pathway mutation status. Bini, binimetinib; BM, biomarker; Enco, encorafenib; Vemu, vemurafenib.

Other gene expression patterns reflecting potentially targetable cell phenotypes were also observed. For example, patients with high *ERBB2* expression showed reduced benefit with encorafenib plus binimetinib versus vemurafenib compared with those with low *ERBB2* expression for PFS [Fig. 2A; PFS HR, 0.60 (95% CI, 0.39–0.92) for high *ERBB2* and 0.36 (95% CI, 0.24–0.55) for low *ERBB2*] and OS [Fig. 2B; OS HR, 0.75 (95% CI, 0.52–1.1) for high *ERBB2* and 0.56 (95% CI, 0.38–0.83) for low *ERBB2*]. Thus, although patients with high *ERBB2* expression still derived a PFS benefit from encorafenib plus binimetinib versus vemurafenib, this was substantially reduced compared with the *ERBB2-*low group, and, unlike in the *ERBB2-*low group, an OS benefit was not significant.

Previous gene expression analyses have found melanoma to segregate into invasive and proliferative phenotypes, differentiated by the expression of *AXL* and *MITF*, respectively (21). In this study, *MITF* and *AXL* expression were negatively correlated across samples (r = −0.44; *P* < 0.001). The benefits of encorafenib ± binimetinib were largely consistent across patients with above versus below median expression of *MITF* or *AXL* (Supplementary Fig. S4A–S4D), although increased *MITF*, but not *AXL*, expression was associated with decreased PFS [HR, 1.44 (95% CI, 1.16–1.79)] and OS [HR, 1.27 (95% CI, 1.03–1.57)] when pooling across arms, with no significant interaction across arms (PFS curves for individual arms shown in Supplementary Fig. S4A and S4C). Expression of AXL was not significantly associated with OS (Supplementary Fig. S4D), and the ratio of *MITF*/*AXL* expression (based on the median of log_2_*MITF* TPM - log_2_*AXL* TPM) was not more strongly associated with outcome than *MITF* expression alone [PFS HR, 1.26 (95% CI, 1.02–1.57); OS HR, 1.26 (95% CI, 1.02–1.55)].

### Gene expression–based molecular subgroups

**Figure 3.**
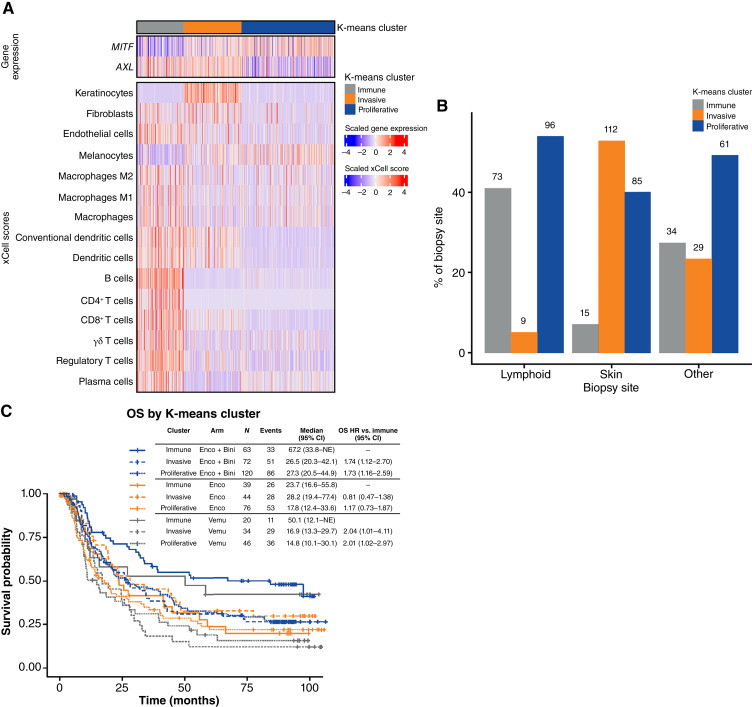
Characterization of tumor samples to identify candidate molecular subtypes by K-means clustering and distribution of subtypes by biopsy site. **A,** Heatmap showing K-means cluster and the expression of key marker genes (*AXL* and *MITF*) and cell type scores generated by xCell. **B,** Distribution of K-means subgroups across biopsy sites. **C,** OS by K-means subgroup and treatment arm. Bini, binimetinib; Vemu, vemurafenib.

To perform unbiased subgroup discovery in COLUMBUS, we used K-means clustering to identify candidate tumor molecular subgroups, with three presumptive subgroups identified (Supplementary Fig. S5). For consistency with prior subtyping systems (21), these were labeled as “immune” based on the expression of immune-related genes (*n* = 122), “invasive” based on low expression of *MITF* and higher expression of *AXL* (*n* = 150), and “proliferative” based on higher *MITF* expression (*n* = 242; Fig. 3A). Interestingly, *AXL* expression was also relatively elevated in the immune subgroup. Estimation of the abundances of key cell types in the TME using xCell (19) corroborated high scores for lymphocytes in the immune subgroup and revealed higher keratinocyte scores in the invasive subtype. The immune subgroup was also significantly associated with biopsy sites (Fisher exact test; *P* < 0.001), with the invasive subtype enriched in skin biopsies and the immune subtype in lymph node metastases (Fig. 3B), thus suggesting gene expression clusters may be representative of biopsy site rather than intrinsic features of the tumors. Furthermore, samples originating from the skin were not classified as primary or metastatic lesions; therefore, it is unclear to what extent the observed differences between skin and other sites reflect the biology of metastatic disease. Nonetheless, differences were observed between the three clusters that were not dependent on biopsy site, and comparative analysis of relative contributions to OS suggested that the gene expression cluster contributed substantially more than biopsy site, with the effect of cluster approximately 28-fold greater. As suggested by preceding analyses, the immune cluster was associated with improved survival compared with the invasive and proliferative clusters, with significantly longer OS for patients with an immune versus an invasive tumor subtype [HR, 0.69 (95% CI, 0.51–0.94); Fig. 3C].

Additional analyses of biomarkers, including cytolytic score and *ERBB2* and *MITF* expression, demonstrated that expression of these cluster-specific features was largely independent of biopsy site (Supplementary Fig. S6). For example, samples in the immune cluster showed higher cytolytic scores than other subtypes across biopsy sites. In contrast, invasive cluster samples had increased *ERBB2 *expression, whereas proliferative cluster samples expressed higher levels of *MITF*. These patterns were largely independent of biopsy site.

To corroborate these targeted findings, we also performed global analyses of gene expression associations with OS, including *z*-scored expression values for each measured gene in univariable Cox models. Consistent with the preceding analyses, key markers of lymphocyte infiltration were associated with improved OS in the encorafenib plus binimetinib arm, with *CD38*, *LAG3*, and *CD8A* among the genes most strongly associated with OS; in contrast, these associations were attenuated in the encorafenib arm alone and largely absent in the vemurafenib arm. In the encorafenib plus binimetinib arm, the expression of *SOX6* was also strongly associated with increased risk. Gene-set enrichment analysis of hallmark gene sets using genes ranked by associations with OS in each arm further corroborated this interpretation, with immune processes (e.g., allograft rejection and IFNγ signaling) dominating the signals recovered from the encorafenib plus binimetinib arm and, to a lesser extent, the encorafenib arm (Fig. 1E).

### PI3K pathway mutations and benefit with encorafenib ± binimetinib

Given the described role of PI3K pathway mutations in resistance to BRAF ± MEK inhibition (22), we tested for associations between pathway alterations and outcome. For patients with mutations in the PI3K pathway, the PFS (Fig. 2C) and OS (Fig. 2D) benefits with encorafenib plus binimetinib versus vemurafenib were reduced compared with patients who were wild-type (WT) for the PI3K pathway [PFS HR, 0.69 (95% CI, 0.40–1.17) for PI3K pathway mutations and 0.51 (95% CI, 0.38–0.69) for WT PI3K pathway; OS HR, 0.83 (95% CI, 0.52–1.33) for PI3K pathway mutations and 0.68 (95% CI, 0.52–0.89) for WT PI3K pathway]. However, in contrast to encorafenib plus binimetinib, encorafenib monotherapy showed numerically longer PFS and significantly longer OS [HR, 0.55 (95% CI, 0.32–0.93)] in patients with PI3K pathway mutations, compared with those without mutations.

### *BRAF* V600 alteration detectability in ctDNA at baseline and on treatment as a prognostic marker for PFS and OS

**Figure 4.**
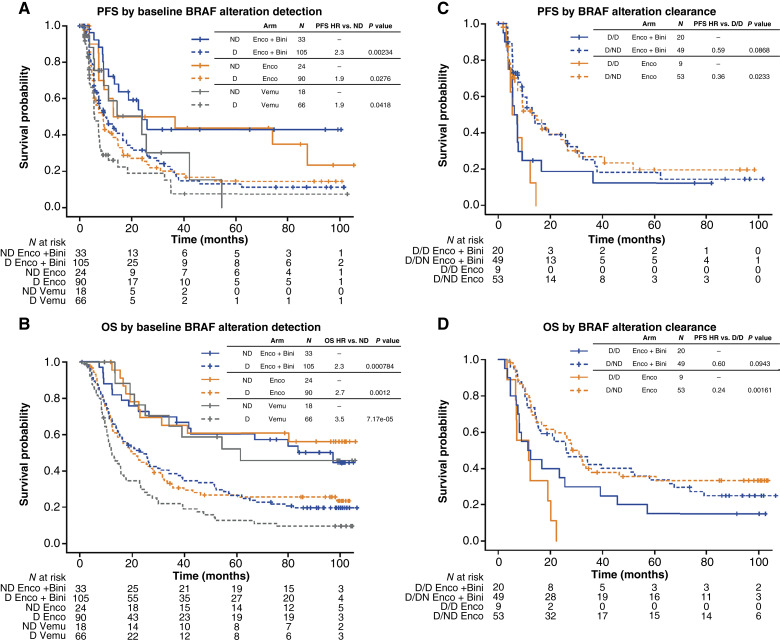
PFS and OS by detection of *BRAF* V600 alterations in ctDNA at (**A** and **B**) baseline and (**C** and **D**) on treatment. Only patients with *BRAF* V600 alteration detections at baseline are shown in **C** and **D**. Bini, binimetinib; D, detectable; Enco, encorafenib; ND, nondetectable; Vemu, vemurafenib.

Detection of *BRAF* V600 alterations in ctDNA at baseline was associated with reduced PFS (Fig. 4A) and OS (Fig. 4B) compared with nondetection, regardless of the treatment arm. This effect was more pronounced for OS (HR of detection vs. nondetection ranged from 2.3 to 3.5 across arms; *P* < 0.01; Fig. 4A) than for PFS (HR, 1.9–2.3; *P* < 0.05; Fig. 4B). On-treatment change in *BRAF* V600 ctDNA detectability was evaluated in the encorafenib and encorafenib plus binimetinib arms and was also prognostic for survival outcomes, despite a limited sample size. For patients with *BRAF* V600 alterations detectable at baseline, those who transitioned to nondetectable at C2D1 of treatment with encorafenib plus binimetinib or encorafenib monotherapy had evidence of improved PFS [HR, 0.36 (*P* = 0.023) in the encorafenib arm; and 0.59 (*P* = 0.087) in the encorafenib plus binimetinib arm; Fig. 4C] and OS [HR, 0.24 (*P* = 0.0016); 0.60 (*P* = 0.094), respectively; Fig. 4D] compared with patients for whom ctDNA remained detectable. Detectability of *BRAF* V600 alterations in ctDNA at baseline was also strongly associated with serum lactate dehydrogenase (LDH) levels. Among ctDNA-evaluable patients with greater than the upper limit of normal (ULN) for LDH (mean, 639.2 U/L in ctDNA-evaluable cases), >99% had a *BRAF* V600 alteration detected, with only a single case with nondetection (*n* = 103/104 detected). Conversely, 68.1% (158/232) of patients with the ULN or lower for LDH (mean, 168.7 U/L) had an alteration detected [*P* < 0.0001 (Fisher exact test)]. As expected, baseline LDH was strongly prognostic for both PFS and OS in ctDNA-evaluable cases, with higher risk in >ULN groups (HR ranged from 2.3 to 4.9 across arms for PFS and 3–3.3 for OS; all *P* < 0.001), with no evidence of differential effects across arms (Supplementary Fig. S7).

To better interpret the relative associations of the above factors with patient outcomes, we examined the correlation among these variables and used recursive partitioning to model OS in the encorafenib and encorafenib plus binimetinib arms. This analysis included LDH, cytolytic score, *BRAF* V600 VAF/detection in baseline ctDNA, TMB, and *MITF* and *ERBB* expression as predictors. Correlations were based on continuous marker values and recursive partitioning based on the cutpoints tested above and included only patients with complete data for these markers. Correlation analysis (*n* = 161 across arms) was consistent with previous observations, with the strongest correlation observed between ctDNA *BRAF* V600 allele frequency and LDH (r = 0.52), followed by more modest negative correlations among gene expression features that reflected previous clustering analyses (Supplementary Fig. S8). Survival analysis largely recapitulated previous univariable analyses, with LDH ranked as the most important factor in both arms; in the encorafenib plus binimetinib arm (*n* = 67), the longest survival was observed in patients with ≤ULN LDH, high cytolytic scores, and high TMB (median OS, 97.4 months; Supplementary Fig. S9), in contrast to patients with >ULN LDH and high *ERBB2* expression (median OS, 10.8 months). In the encorafenib arm (*n* = 54), the longest survival was observed in patients with ≤ULN LDH and low *MITF* expression (median OS, 46.4 months), in contrast to patients in the >ULN LDH group (median OS, 9.89 months).

## Discussion

Exploratory analyses based on the 7-year follow-up of the COLUMBUS study indicate that survival benefits from encorafenib plus binimetinib were observed across most molecular subgroups in *BRAF* V600E/K–mutant melanoma, and no subgroups showed inferior outcomes when treated with encorafenib plus binimetinib versus vemurafenib. Benefits with encorafenib monotherapy versus vemurafenib were less pronounced than with encorafenib plus binimetinib, supporting the use of the BRAF inhibitor ± MEK inhibitor combination over a single-agent BRAF inhibitor.

The PFS and/or OS benefits with encorafenib plus binimetinib versus vemurafenib were greater among patient subgroups with evidence of potential for antitumor immune response at study entry (e.g., higher TMB, cytolytic score, PD-L1 expression, and IFNγ gene expression signature scores) compared with subgroups with low levels of these biomarkers. Notably, although PFS was improved with encorafenib plus binimetinib versus vemurafenib regardless of immune-related biomarker levels, an OS benefit was only observed for patients in the high TMB, cytolytic score, PD-L1 expression, and IFNγ gene expression signature groups.

In contrast to the aforementioned patient subgroups, patients with PI3K pathway mutations seem to have a smaller benefit with encorafenib plus binimetinib, but this benefit remains superior to vemurafenib. As has been previously suggested (22), this suggests a potential role for the PI3K pathway in intrinsic or acquired resistance to encorafenib plus binimetinib. Dysregulation of the PI3K pathway as a factor contributing to acquired resistance to BRAF inhibitors in melanoma has been documented in previous studies (23, 24). It was notable that the same effect was not observed with encorafenib monotherapy, in which patients with PI3K pathway mutations instead seemed to have improved outcomes. The driver of this potential disparity remains unclear, and additional work with larger sample sizes will be required to confirm the effect. The reduced treatment benefits over vemurafenib in patients with mutations in the PI3K pathway may support a potential role for the PI3K pathway in resistance to treatment that warrants follow-up.

Reduced survival benefits with encorafenib plus binimetinib versus vemurafenib were also observed for patient subgroups with high *ERBB2* (HER2) expression, compared with those who had low *ERBB2* expression, suggesting a potential role *ERBB2* in resistance to encorafenib plus binimetinib. Melanoma is not typically considered to be a candidate for HER2-targeting therapies due to substantially lower HER2 positivity rates and expression levels than observed in cancers that are commonly HER2-amplified (25). However, expression (measured via RNA-seq) was elevated in invasive subgroups, and potential for synergistic therapies that target lower levels of expression may be possible. Resistance associated with HER2 overexpression may also be mediated by downstream PI3K pathway activation (26). Resistance to BRAF inhibitors remains a clinical problem in the BRAF V600E/K–mutant metastatic melanoma landscape (23), and patients with PI3K pathway mutations or those with higher *ERBB2* expression may benefit from the addition of other therapies to encorafenib plus binimetinib.

The identification and characterization of tumor subtypes—immune, invasive, and melanocytic/proliferative—based on gene expression and biomarker analyses are consistent with prior classification of melanomas as invasive versus proliferative and further delineate a subgroup with markedly higher immune infiltration. Consistent with the analysis of individual biomarkers, the immune subtype demonstrated the longest OS. Associations between proliferative and invasive melanoma markers (27), such as *MITF* and *AXL*, respectively, and survival outcomes suggest that melanocyte plasticity may influence therapeutic response. The overall negative prognostic impact of high levels of *MITF* on PFS and OS across the encorafenib plus binimetinib, encorafenib monotherapy, and vemurafenib treatment arms is consistent with previous suggestions that *MITF* expression is associated with resistance to targeted therapy (28).

The observation that encorafenib plus binimetinib resulted in a more pronounced treatment benefit versus vemurafenib in patients with tumors with evidence of immune infiltration is consistent with previous biomarker analyses that reported a benefit of BRAF inhibitor ± MEK inhibitor combinations for patients with BRAF-mutant melanoma with an elevated IFNγ signature and an immune signature that included *GZMA* (a component of the cytolytic score), among other genes (29, 30). It also parallels a beneficial effect of a high cytolytic score observed in patients with BRAF-mutant colorectal cancer treated with encorafenib plus binimetinib (plus cetuximab) in the BEACON study (31), suggesting that immunologic correlates of benefit from encorafenib plus binimetinib may be observable in multiple cancer types.

Whereas patients historically received BRAF inhibitor plus MEK inhibitor combinations as first-line therapy for BRAF V600–mutant melanoma, treatment paradigms have fundamentally shifted following recent clinical trial results. The DREAMseq trial showed superior outcomes with immunotherapy-first approaches, with 2-year OS of 72% versus 52% for targeted-therapy-first approaches (32). Current practice guidelines now recommend immunotherapy as first-line treatment for most patients with BRAF V600–mutant melanoma, reserving BRAF/MEK inhibitors for second-line therapy after immunotherapy failure or in specific clinical scenarios requiring rapid disease control (8). Our results suggest that an immune-infiltrated TME may lead to increased benefit from encorafenib plus binimetinib, which might be potentiated by prior immunotherapy. However, the SECOMBIT study found numerically lower response rates to encorafenib plus binimetinib after progression on nivolumab plus ipilimumab than in first-line therapy, suggesting that other factors may overcome a priming effect of immunotherapy, if present (33). Due to the era of this study, very few patients had been previously treated with immune checkpoint blockade, and we were unable to robustly evaluate this possibility in this study (4, 17). Furthermore, similar preclinical and clinical findings suggesting that BRAF inhibitors and/or MEK inhibitors engage or exploit the immune system have led to multiple (34) phase II and III trials, including COMBI-I, IMspire150, and KEYNOTE-022, that explored the combination of BRAF inhibitors/MEK inhibitors and immune checkpoint blockade. The results of these studies have so far failed to lead to a recommendation of combination triplet therapies for BRAF-mutant melanoma in routine clinical practice (4, 35, 36). Observations of the expected immunostimulatory effects of MEK inhibition alone have led to trials such as IMspire170, which combined an MEK inhibitor (cobimetinib) with immune checkpoint blockade in *BRAF*-WTmelanoma, but failed to demonstrate clear benefit. The ongoing STARBOARD and PORTSIDE studies combine encorafenib and binimetinib with pembrolizumab or nivolumab plus ipilimumab, respectively, and may help clarify the potential for immune synergies suggested (37, 38). The detectability of *BRAF* V600 alterations in ctDNA at baseline and changes in detectability during treatment were associated with PFS and OS across treatment arms, with effect sizes largely consistent with those observed in a meta-analysis of metastatic melanoma studies (39). Detection of *BRAF* V600 alterations at baseline was highly correlated with serum LDH, but alterations were still frequently detectable in patients with LDH below the ULN, suggesting potential value for ctDNA profiling in patients with lower LDH levels. Thus, these results are in accord with the use of ctDNA as a marker of survival and/or response in BRAF V600E/K–mutant advanced or metastatic melanoma that warrants further validation.

This analysis pooled data from the encorafenib plus binimetinib arms in COLUMBUS part 1 (encorafenib 450 mg once daily) and part 2 (encorafenib 300 mg once daily), which limits conclusions regarding encorafenib dosing in the combination regimen and potentially biomarker comparisons among arms in this study. Furthermore, as this was a retrospective analysis, it was not able to address all hypotheses of interest with sufficient or equivalent power, and further prospective analyses are needed to validate the findings proposed here. These analyses corroborated a greater benefit of encorafenib plus binimetinib versus vemurafenib across genetic and transcriptional biomarker subgroups and did not identify any subgroups of patients who had improved outcomes with vemurafenib versus encorafenib plus binimetinib. Increased benefit was most notable in tumors with evidence of immune infiltration or potential for antitumor immune response (e.g., with elevated TMB), thus supporting the hypothesis that encorafenib plus binimetinib might show synergistic effects when combined with immunotherapies.

In conclusion, survival benefit from encorafenib ± binimetinib was observed across the majority of molecular subgroups, and this benefit was most pronounced in patients treated with encorafenib plus binimetinib with heightened signatures of immune infiltration and elevated TMB.

## Supplementary Material

Supplementary Data Supplementary Data

## Data Availability

Upon request, and subject to review, Pfizer will provide the data that support the findings of this study. Subject to certain criteria, conditions, and exceptions, Pfizer may also provide access to the related individual deidentified participant data. See https://www.pfizer.com/science/clinical-trials/trial-data-and-results for more information and for instructions on how to submit a request.
