# Supplementary material for: Exploratory Analysis of Biomarkers and Treatment Outcomes from the COLUMBUS Study in BRAF V600E/K–Mutant Advanced or Metastatic Melanoma
Source: Clin Cancer Res. 2026 Jan 15;32(7):1266–76. doi: 10.1158/1078-0432.CCR-25-3262 (PMC13040209; doi:10.1158/1078-0432.CCR-25-3262)

**SUPPLEMENT**

**Figure S1. Study design for COLUMBUS Part 1 and Part 2.**


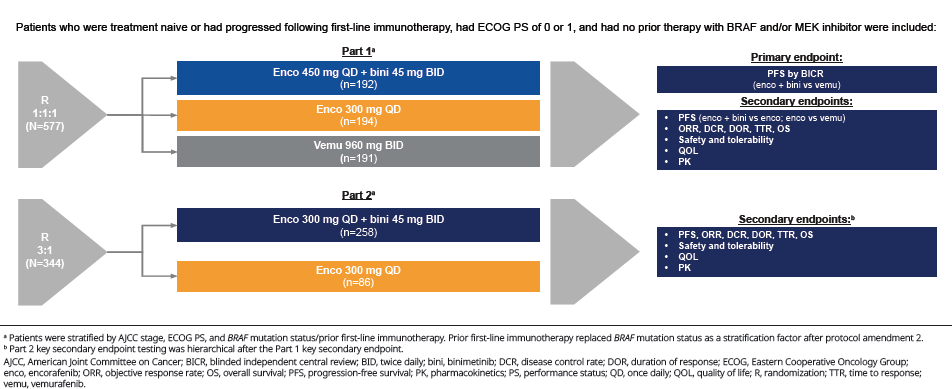


**Figure S2. WES, RNA-seq, and ctDNA collection from COLUMBUS.**

Abbreviations: bini, binimetinib; C, cycle; ctDNA, circulating tumor DNA; D, day; enco, encorafenib; vemu, vemurafenib; RNA-seq, RNA sequencing; WES, whole exome sequencing.

**
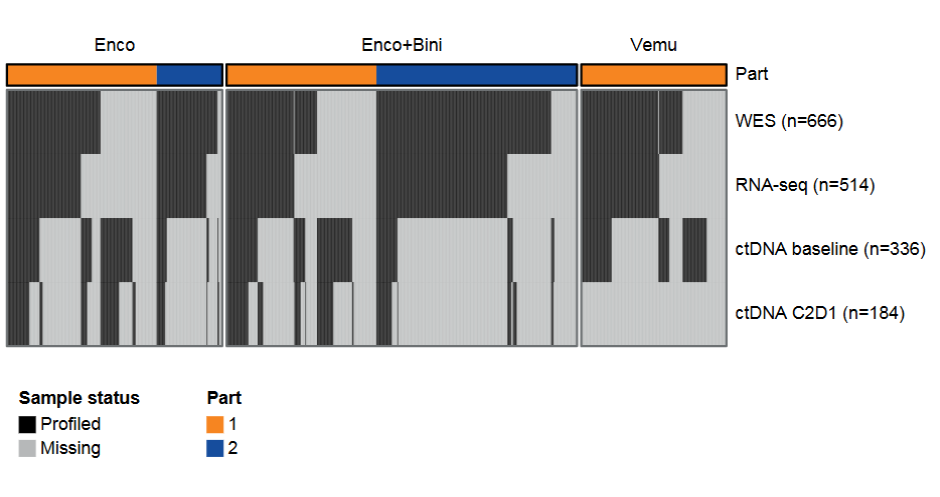
**

**Figure S3. PFS and OS for encorafenib plus binimetinib or encorafenib versus vemurafenib by (a, b) PD-L1 expression level and (c, d) IFNγ signature. (e) Correlation plots for cytotoxic score,** **IFNγ signature, and PD-L1 expression level.**

Abbreviations: bini, binimetinib; BM, biomarker; CYT, cytotoxic score; enco, encorafenib; IFNγ, interferon gamma signature; OS, overall survival; PFS, progression-free survival; TMB, tumor mutational burden; vemu, vemurafenib.

**
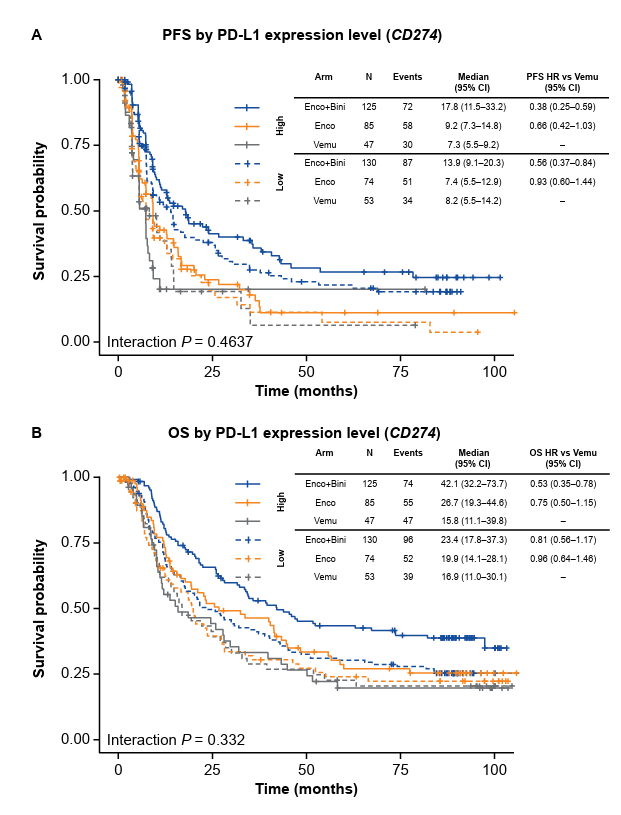
**

**
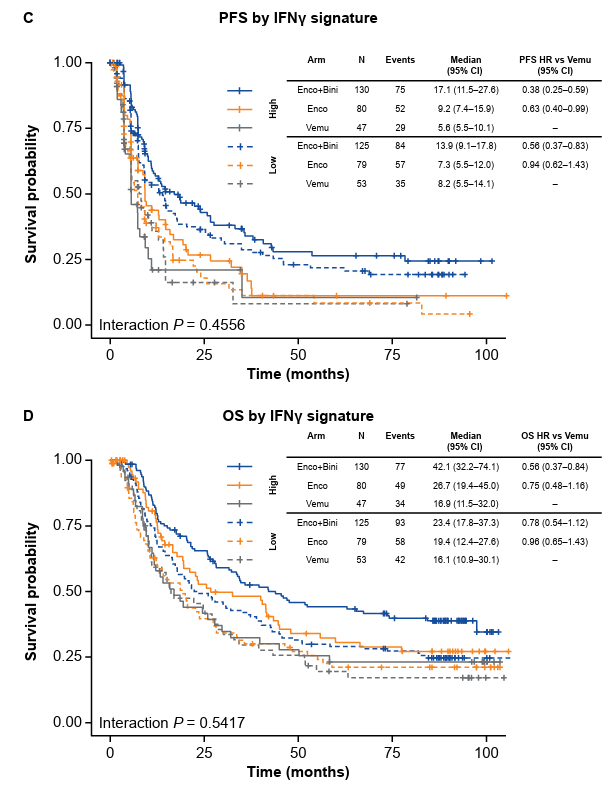
**

**
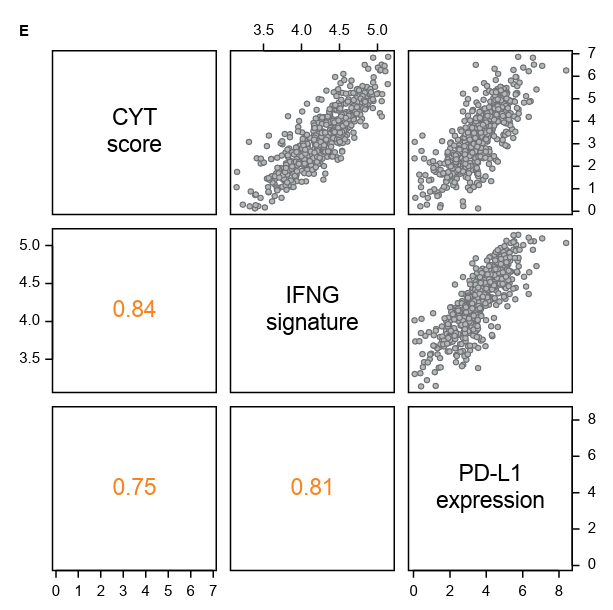
**

**Figure S4. PFS (a) and OS (b) based on *MITF* expression. PFS (c) and OS (d) based on *AXL* expression. Expression subgroups are defined based on median *MITF* expression.**

Abbreviations: bini, binimetinib; enco, encorafenib; HR, hazard ratio; LDH, lactate dehydrogenase; OS, overall survival; PFS, progression-free survival; trt, treatment; vemu, vemurafenib.**
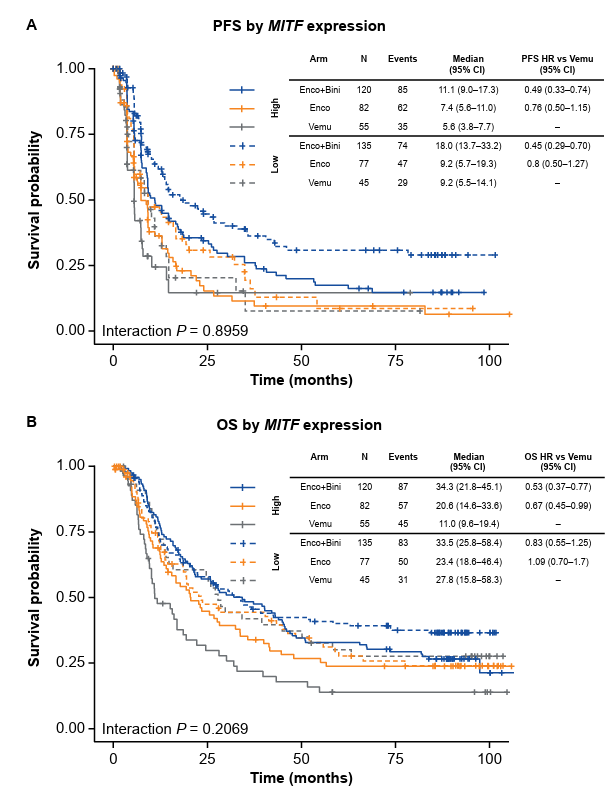
**

**
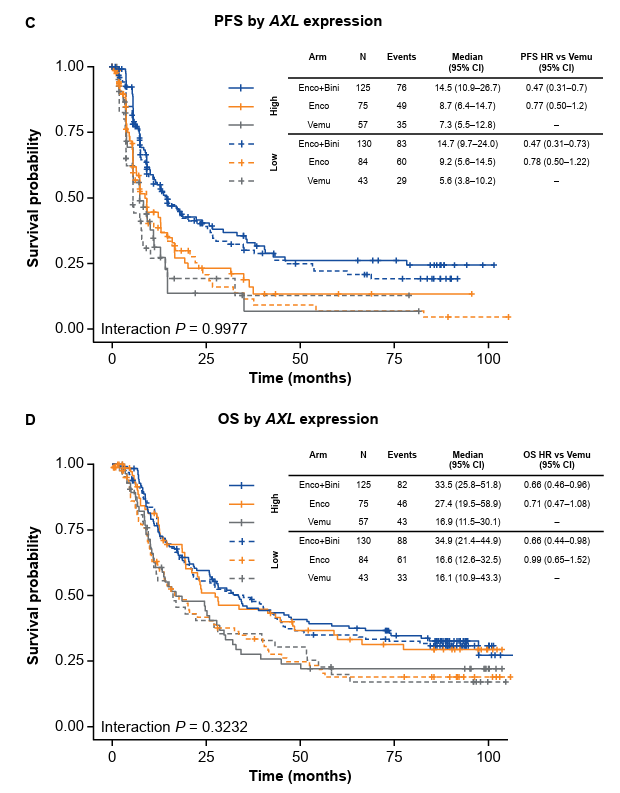
**

**Figure S5. Clustering In RNA-seq data.**

**
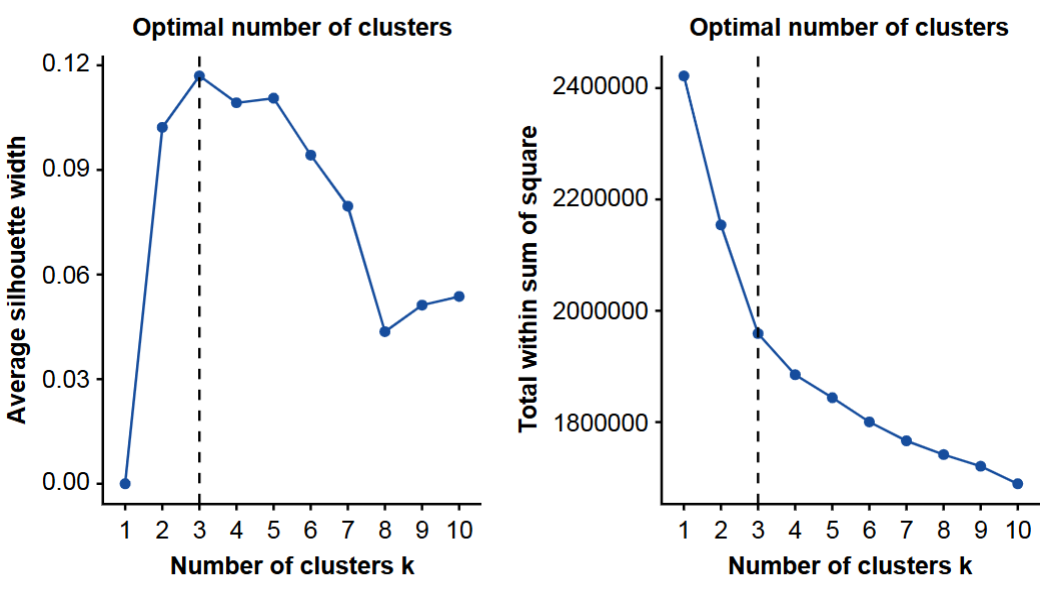
**

**Figure S6. Expression of key markers by cluster and biopsy site.**

Abbreviation: TPM, transcript per million.


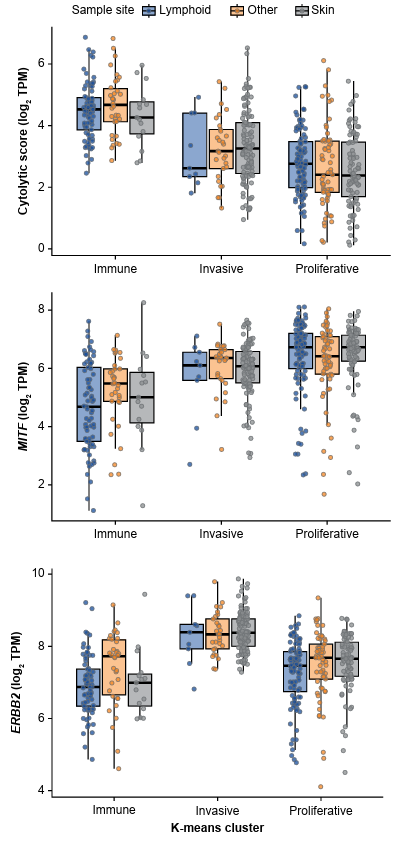


**Figure S7. PFS (a) and OS (b) by baseline LDH status.**

Abbreviations: bini, binimetinib; enco, encorafenib; HR, hazard ratio; LDH, lactate dehydrogenase; OS, overall survival; PFS, progression-free survival; ULN, upper limit of normal; vemu, vemurafenib.


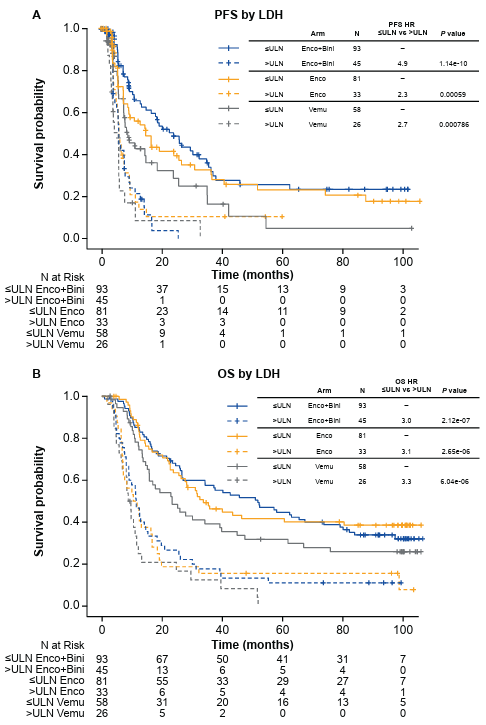


**Figure S8:** Correlation of features implicated in benefit to encorafenib ± binimetinib. Patients with complete data across all features irrespective of arm were included (n = 161). Pearson correlations are shown. The absence/presence of PI3K pathway mutations was coded as 0/1 for analysis.


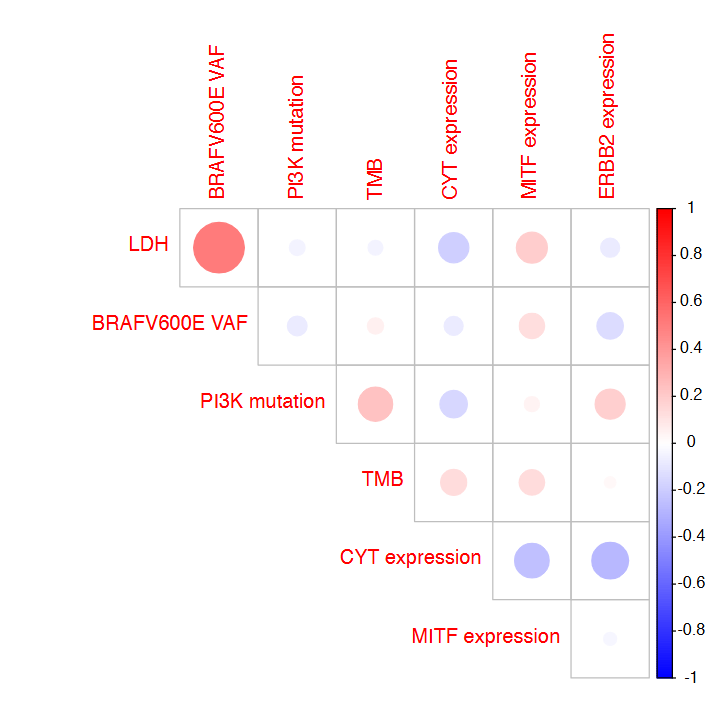


**Figure S9. Recursive partitioning analysis.** Models were fit within the A) encorafenib arm and B) encorafenib + binimetinib arm separately. Analysis was restricted to cases with complete data across tested predictor variables, with overall survival as the outcome (shown in lower Kaplan–Meier plots). Binary cut-points for continuous variables are based on median values unless otherwise noted. Numbers on inner nodes represent node positions in the partitioning tree.


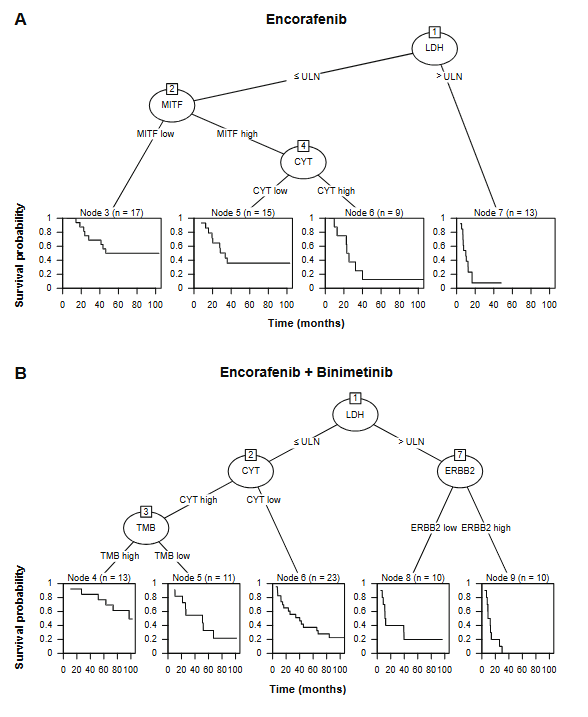

Supplement: Supplementary Data [file ccr-25-3262_supplementary_data_suppsd.docx]
